# 2613. Pediatric respiratory syncytial virus hospitalizations, 2017 to 2022, the Canadian Immunization Monitoring Program Active (IMPACT)

**DOI:** 10.1093/ofid/ofad500.2226

**Published:** 2023-11-27

**Authors:** Malou Bourdeau, Nirma K Vadlamudi, Christina Bancej, Nathalie Bastien, Joanne Embree, Scott Halperin, Andrea Hudgin, Taj Jadavji, Kescha Kazmi, Joanne M Langley, Marc Lebel, Nicole Le Saux, Dorothy L Moore, Shaun Morris, Jeffrey Pernica, Joan Robinson, Manish Sadarangani, Julie A Bettinger, Jesse Papenburg

**Affiliations:** McGill University, Montréal, Quebec, Canada; Department of Pediatrics, The University of British Columbia, Vancouver, British Columbia, Canada; Center for Immunization & Respiratory Infectious Diseases, Public Health Agency of Canada, Ottawa, Ontario, Canada; National Microbiology Laboratory, Public Health Agency of Canada, Winnipeg, Manitoba, Canada; Department of Pediatrics, University of Manitoba, Winnipeg, Manitoba, Canada; IWK Hlth Ctr, Halifax, NS, Canada; Canadian Center for Vaccinology, IWK Health Center, Dalhousie University, Halifax, Nova Scotia, Canada; Alberta Children’s Hospital, University of Calgary, Calgary, Alberta, Canada; Hospital for Sick Children, University of Toronto, Toronto, Ontario, Canada; Dalhousie University, IWK Health and Nova Scotia Health, Halifax, Canada, Halifax, Nova Scotia, Canada; Department of Pediatrics, Centre hospitalier universitaire Sainte-Justine, Montreal, Quebec, Canada; Children's Hospital of Eastern Ontario, Ottawa, ON, Canada; Department of Pediatrics, McGill University, Montreal, Quebec, Canada; Hospital for Sick Children, University of Toronto, Toronto, Ontario, Canada; Department of Pediatrics, McMaster University, Hamilton, ON, Canada; Stollery Children's Hospital, Edmonton, AB, Canada; University of British Columbia, Vancouver, British Columbia, Canada; University of British Columbia, Vancouver, British Columbia, Canada; Departments of Pediatrics and Medical Microbiology, McGill University Health Centre, Montreal, QC, Canada, Montreal, Quebec, Canada

## Abstract

**Background:**

Respiratory syncytial virus (RSV) is a leading cause of pediatric hospitalizations. We aimed to describe the epidemiology and burden of RSV-associated hospitalizations among children in Canadian pediatric centers from 2017 to 2022, including changes during the COVID-19 pandemic.

**Methods:**

We performed active surveillance for hospitalized children 0 to 16 years of age with laboratory confirmed RSV at 13 Canadian Immunization Monitoring Program Active (IMPACT) pediatric hospitals during 5 seasons (2017-18 to 2021-22). Proportions of RSV hospitalizations over all-cause hospitalizations over time, and intensive care unit (ICU) admissions, prolonged admissions (≥ 7-days) and mortality proportions were calculated, overall and by age groups and regions. RSV hospitalization-associated burden was compared for 2021-22 to the pre-pandemic period of 2017-18 to 2019-20. Seasonality was described using epidemic curves.

**Results:**

Among 11,014 RSV-associated hospitalizations 6,035 (54.8%) were male and 5,488 (50%) were aged < 6 months. Overall, 2,594 (23.6%) were admitted to ICU, of which 60.8% were aged < 6 months old. The median hospital stay was 4 days (interquartile range: 2-6). The mean number of hospitalizations during the pre-pandemic seasons was 2,522. Only 58 cases were reported in 2020-21, followed by 3,170 in 2021-22. The proportion of RSV hospitalizations over all-cause hospitalizations rose from 3.2% pre-pandemic to 4.5% in 2021-22 (difference 1.3% [95%CI 0.8-1.8]; p=0.07 after multiplicity adjustment). One province, Quebec, had a significant increase in RSV-hospitalization proportion in 2021-22 (2.5 percentage points, 95%CI 1.7-3.2, adjusted p-value 0.045). Age, sex, ICU admission, prolonged length of stay(≥7-days) and mortality proportions did not change in 2021-22 compared to the pre-pandemic period. Interregional differences in RSV seasonality were accentuated in 2021-22.

Weekly RSV-associated hospital admissions in children aged 0 to 16 years at IMPACT centers, 2017-2022, by season

**Figure:**
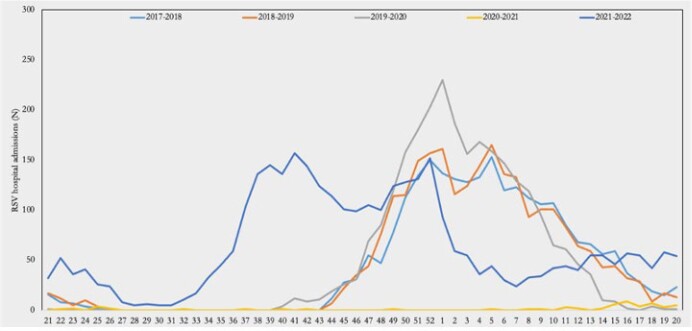

Monthly RSV-associated hospital admissions in children aged 0 to 16 years at IMPACT centers, 2017-2022, by province

**Figure:**
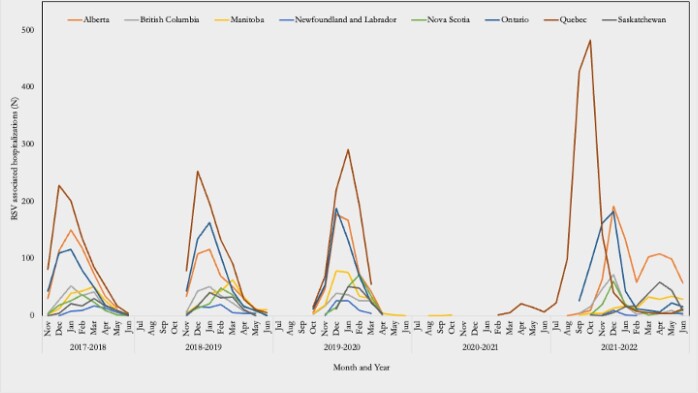

**Conclusion:**

RSV hospitalization burden in Canadian pediatric hospitals is substantial, especially in infants aged < 6 months. Following a near absence in 2020-21, RSV hospitalizations increased in the 2021-22 season, but severity of illness remained similar to the pre-pandemic period. These data will aid planning of RSV prevention strategies.

**Disclosures:**

**Nirma K. Vadlamudi, MPH, PhD**, Broadstreet HEOR: Personal fees outside of the submitted work **Scott Halperin, MD**, CanSino: Grant/Research Support|CanSino: served on ad hoc advisory board|GlaxoSmithKline: Grant/Research Support|GlaxoSmithKline: served on ad hoc advisory board|Merck: Grant/Research Support|Merck: served on ad hoc advisory board|Moderna: Grant/Research Support|Moderna: served on ad hoc advisory board|Pfizer,: Grant/Research Support|Pfizer,: served on ad hoc advisory board|Sanofi-Pasteur: Grant/Research Support|Sanofi-Pasteur: served on ad hoc advisory board|Seqirus: Grant/Research Support|Seqirus: served on ad hoc advisory board|VBI Vaccines: Grant/Research Support|VBI Vaccines: served on ad hoc advisory board **Joanne M. Langley, MD**, CanSino: Grant/Research Support|Entos: Grant/Research Support|GlaxoSmithKline: Grant/Research Support|Merck: Grant/Research Support|Moderna: Grant/Research Support|Pfizer: Grant/Research Support|Sanofi-Pasteur: Grant/Research Support|Seqirus: Grant/Research Support|Symvivo: Grant/Research Support|VBI Vaccines: Grant/Research Support **Shaun Morris, MD, MPH, DTM&H, FRCPC, FAAP**, GlaxoSmithKline: Honoraria|JNJ China: Honoraria|Merck: served on ad hoc advisory board|Pfizer: Grant/Research Support|Pfizer: served on ad-hoc advisory board|Sanofi-Pasteur: served on ad-hoc advisory board **Jeffrey Pernica, MD, MSc, FRCPC, DTMH**, MedImmune: Grant/Research Support|Merck: Grant/Research Support **Manish Sadarangani, BM BCh, FRCPC, DPhil**, GlaxoSmithKline: Grant/Research Support|Merck: Grant/Research Support|Moderna: Grant/Research Support|Pfizer: Grant/Research Support|Sanofi Pasteur: Grant/Research Support|Seqirus: Grant/Research Support|Symvivo: Grant/Research Support|VBI Vaccines: Grant/Research Support **Jesse Papenburg, MD**, AstraZeneca: Personal fees outside of the submitted work|MedImmune: Grant/Research Support|Merck: Grant/Research Support|Merck: Personal fees outside of the submitted work

